# Feeling and Thinking about It Are Two Different Things: How to Capture Momentary Emotions of Extreme Sports in the Field

**DOI:** 10.3390/ijerph19031290

**Published:** 2022-01-24

**Authors:** Audun Hetland

**Affiliations:** Department of Psychology, Faculty of Health Sciences, UiT the Arctic University of Norway, 9037 Tromsø, Norway; audun.hetland@uit.no; Tel.: +47-93041612

**Keywords:** emotions, facial recorded emotions, extreme sport, face reader, self-report

## Abstract

To learn about extreme sports and what motivates such activities, we need to understand the emotions embedded in the experience itself. However, how we go about assessing these emotions might provide us with very different answers. An experience is a fleeting and ever-changing phenomenon, rich in detail and filled with nuances. What we remember and, therefore, what we are able to report from our experience might, however, be strikingly different to what we experienced. Our memories are grained by time, impaired by arousal, and affected by context. Despite these limitations, the most common way to measure an experience is by self reporting. The current paper reviews some of the relevant theory on emotions and how this might impact different assessments. I also describe a new way of measuring momentary emotions in the field by use of video cameras and automatic coding of facially expressed emotions. Extreme sports may leave us with positive memories but may be anything but pleasant while in the midst of them. In the end, this paper may give some hints to why.

## 1. Introduction

He is scared. Even the baggy clothes cannot hide his shivering while he takes the last microscopic step and places his toes on the very edge of the cliff. Between his toes and the bottom of the valley is a whole lot of nothing, 1003 meters of nothing, to be exact. The weather is perfect. No wind, just sun and an exceptionally pleasant temperature. We could have spent this perfect day relaxing on the beach. Instead, we are here, far from most of the stuff that matters in a normal life. “Are you ready to jump?” One of the experienced BASE jumpers looks at the owner of the toes, as unaffected as if he just asked him to step on board a bus. The owner nods. I look down into the viewfinder of my camera, making sure that I get everything on film. As the owner takes a last deep breath, one word keeps on bouncing around in my mind: why?

A range of studies describe a multitude of motives for taking part in extreme sport [1,2,3,4,5,6,7,8]. Some of the divergence may be due to the challenges of defining what extreme sport really is, and what types of activity sort under the extreme sport umbrella [9,10]. However, at its core, humans tend to repeat what makes them feel better and avoid what makes them feel worse [11]; in the midst of it, however, such experiences seem to be everything but pleasant [5,6,8]. What is it with these experiences that makes them so captivating that they lead people to repeat them over and over again? In an attempt to understand, we need to explore the emotional experience during such activities and track how these experiences are transformed into positive memories. In earlier studies on backcountry riders [6] and mountain bikers [8], we measured participants facially expressed emotions from moment-to-moment and compared the results to the participants’ self-reported emotions. The results from these studies show that there is an apparent shift within the momentary measures between when the participants are in the midst of their activity and when they pause. There is also a major difference between moment-to-moment facially expressed emotions and retrospective self-reported emotions obtained immediately after the activity is over. Given the variation within a single extreme sport episode such as a BASE jump, what you measure, how you measure and when you measure will provide different results.

In this paper, I will describe in more detail how facially expressed emotions can be used to measure momentary emotions in the lab and in the field. I will also address some challenges with this method and ordinary self reporting. Efforts to measure the emotional quality during and after the event might help explain how such intense experiences turn into pleasant memories that motivate the participants to repeat the endeavor. To understand this, we need to start with a short review of emotions, their functions and how they may change over the course of an extreme sport episode. 

### 1.1. Emotion as a Motive for Taking Part in Extreme Sport

In the first half of the 1900s, voluntary risk-taking was seen as evidence of pathology [12,13]. Later, extreme sport athletes have been portrayed as egocentric risk-seeking individuals, and scholars have provided different theoretical explanations to the apparent abnormality in partaking in extreme sport, including: sensation seeking [14,15], psychoanalysis [16], type T personality [17], reversal theory [18], and edge work [19]. For a review, see Brymer and Mackenzie [20]. However, as noted by Milovanovic [21], many of the traditional explanations are simplistic and based on a naïve non-participant point of view.

The extreme sport participants themselves describe their effort to hone their skills and minimize risk [22]. Storry [23] noted that the focus on risk and thrill misses the point entirely.

Recent studies, in contrast, describe growth-oriented motives such as mastery, peak experiences, transcendence and flow, wherein the participants are able to acquire increasing levels of mastery and skills in activities that produce often otherwise-inaccessible feelings of joy and elation [1,4,5,6,8,24,25,26,27]. Such experiences do not exist in a vacuum but rather constitute a dynamic interplay between the athletes and the environment, their behavior and psychological expressions [28], and affected by their personal and social identities [29]. However, at its core is the emotional experience itself.

Measuring these emotions is not an easy task. There exists a wide range of measures, from simple self-reports, sampling immediate or reconstructed experiences, to complex physiological measures of bodily processes and behavior. These measures assess different components of an emotional experience at different times, and will therefore often produce different results. Before I describe some of these methods, we need to review some of the important facets of emotions.

### 1.2. What Is an Emotion?

Feelings and emotions are not the same. As reviewed in Vittersø [30], feelings are both broader and narrower than emotions. They are broader in the sense that as long as we are conscious, we always feel something. This general feeling state reflects what it is like to have a subjective experience, such as eating a banana or seeing a red tomato, but these feelings need not be emotional [31]. However, feelings are also narrower than emotions, because they are just one among several components of an emotion. In addition to subjective experience, emotions consist of components such as physiological processes, bodily expressions, and action readiness [32]. In this paper, I will use feelings as a description of the subjective experience of an emotion, while emotions will be used as a description of the coherent package of emotional components, where feelings are one component.

There are two major and competing classifications of emotions: the dimensional approach and the discrete or basic emotions approach [33,34]. From a dimensional point of view, a few broad dimensions such as pleasure and arousal are the fundamental building blocks of emotions. Emotions can further be comprised into two broad categories of positive and negative affect [35,36,37]. From a basic emotions point of view, there exists a handful of discrete emotions, which are fundamentally different from one another. These are seen as innate and hard-wired and cannot be divided further into elements that are not emotions [38,39].

Emotions have functions, and according to Oatley [38], each of these basic emotions activates a predefined set of cognitive “checklists” and primes us for a set of actions. The feeling of fear, evoked from a threatening situation, will set up our cognitive system to confront the danger. As for the physiological component of emotions, our body will be set in a fight or flight mode with increasing heart rate and blood pressure. The behavioral expressive component will produce facial expressions of fear and our vocal pitch and body posture will change [40,41,42]. The action readiness component will terminate all ongoing activity, and we will focus solely on the source of danger, while we prepare for and execute appropriate counter action.

#### 1.2.1. Two Levels of Emotional Experience

There are two types or levels of emotional experience: a continuous and sometimes unconscious stream of raw feelings (first level) and an accompanying reflective cognitive overlay (second level). The continuous flow of raw feelings typically includes feelings and desires, many of which include physiological components and felt action urges. The reflective cognitive overlay includes conscious thoughts, appraisals of emotional states, or the environment. Although raw feelings and reflective cognition might be identical, they often diverge. What determines the balance between them is, as Nilsen and Kaszniak [43] put it, how much one is “in the grip” of emotion. 

Putting experiences into words demands a level of cognitive reflection [44]. If people are totally immersed, they will be unable to report their emotions. When they regain the ability to report, their self-reported emotions will, consequently, be a mix between raw feelings and reflective cognition, leaving the pure raw feelings beyond reach.

Such unconscious emotions can be powerful enough to affect behavior without people ever becoming consciously aware of it happening [45]. However, a lack of access to these first-level emotional experiences might lead people to infer what they feel based on self-observation, much like an outside observer [46]. Indeed, split-brain patients have reported second-order emotional awareness without any first-order emotional experiences ever occurring [47]. Wiens [48] proposes a hypothetical model wherein second-level awareness is based on either first-level emotional experiences or actual or illusory perceptions of physiological changes.

A slightly different view on the distinction between moment-to-moment and overall emotions is presented in the functional wellbeing approach (FWA [49,50]). The FWA argues that momentary emotions are generated by the step-by-step execution of small acts that lead towards the goal. As such, overall emotions arise as an evaluation of goal accomplishment for the whole event. If the goal is successfully reached, this will evoke emotions such as pleasure or satisfaction. If the goal is not achieved, this will lead to negative emotions such as sadness or anger. Therefore, when we assess people’s emotions (e.g., during or after an activity) may fundamentally impact the result we receive. A difficult activity could, for example, be reported as interesting or even frustrating while in the midst of it, but if the task is successfully accomplished, happiness is likely reported in retrospect. Similar findings are also described in the field of exercise psychology, wherein pleasure declines during exercise followed by a positive rebound that exceeds the baseline after the activity is over [11]. This is in line with the results from several of our previous studies where we found that there is little correlation between the moment-to-moment measures of facially expressed emotions and the retrospective self-reported emotions [6,8]. We even find the same pattern within the activity itself where backcountry skiers [6] do not express any happiness while skiing but display increasing levels of happiness as soon as they start to slow down (as an example of this shift, see the film from the analysis of backcountry skiing participants [51].

In an ongoing mixed-method study of ski flyers, the participants describe the shift from being fully immersed and also experiencing high levels of fear at the start of their jump to happiness towards the end of flight and after landing. We find the same pattern in a case study reported in this paper from one of the jumpers wearing a face-fronting camera [52]. The optimal or dysfunctional impact of such emotions on athletes’ performance depends not only on the emotional content but also on their intensity [53]. This leads us to another set of inherent challenges in extreme sport, namely the high levels of high arousal and its impact on the ability to report one’s emotions.

#### 1.2.2. Arousal

The common understanding of high arousal is to be wide awake, excited, vigorous, and alert. To be unaroused means to be relaxed, sleepy or tired [54,55]. The level of arousal may be measured in a variety of ways, from cortical activity such as EEG measures to autonomic measures such as skin conductance (SC) and heart rate (HR) [55]. Although there is evidence that arousal consists of a range of separate arousal systems, McGaugh [56] has shown how separate arousal systems serve the same function, which is to moderate the resources available for information processing [54].

On the one hand, high arousal at encoding facilitates both detection and encoding for long-term retrieval. On the other hand, it may also lead to an inability to retrieve information for a short period of time, up to 30 min, after the original experience [54]. For example, in their well-known article, Kleinsmith and Kaplan [57] found that arousing words were better remembered after one week than they had been two minutes after learning. This is in line with research on stress hormones, in which high arousal has been shown to increase the ability to retrieve information in the long run [58].

High arousal has also been shown to cause an increasing number of false memories [59]. Given the effect arousal has on immediate retrieval, self-reported emotions may be severely biased if administered immediately after an extreme sport experience (for further arguments, see [59]. However, a delayed report of emotional states creates yet another set of problems, namely what we are able to remember.

#### 1.2.3. Memory

Our memory is a far-from-perfect representation of the actual event, and this is also true for our remembered emotions. The more time between the experience and the report of it, the greater the chance for errors or biases. Robinson and Clore [60] argue that people prefer to use the most specific source of information when reporting their emotions. They suggest that people access at least four types of knowledge to assess their emotion. Ranging from most to least specific, they are: experiential knowledge, episodic memory, situation-specific belief, and identity-related belief. 

Experiential knowledge is the direct access of current emotions. This information can neither be stored nor retrieved. However, through episodic memory, people can attempt to retrieve specific moments or contextual details from the past. Although past emotional experiences cannot be re-experienced, they can often be reconstructed, aided by such memory cues. 

Situation-specific beliefs are people’s belief that certain emotions are likely to be experienced in a particular type of situation. For instance, most of us believe that vacations are associated with happiness and the death of a loved one with sadness. Finally, identity-related beliefs are the beliefs people hold onto their emotional experiences in general, such as their emotional traits, but also normative social beliefs. One of the most prominent beliefs is a gender stereotype in which people believe that women are more emotional than men. Indeed, in retrospective reports, women report higher levels of emotion than men [61,62]. However, these differences seem to disappear when measured as the activity unfolds [60]. All of these four sources give potentially different information about the individual’s emotional experiences. This shows how sensitive self-reports and questionnaires can be (see also [61]), depending on the kind of information being accessed.

## 2. Some Challenges with Measuring Emotional Experiences

### 2.1. Self-Reports

When I planned the study on BASE jumpers, I had no tool for measuring momentary emotions. I eventually ended up with a design inspired by experience sampling [62] and day reconstruction [63]. To sample the BASE jumpers’ experiences, I asked the jumpers to fill out a questionnaire immediately after landing. However, high levels of arousal are shown to impair participants’ reporting [54]. I therefore wanted to reconstruct the experience in a low-arousal setting. To aid their recall of the experience in a low-arousal setting, I showed them a film of their jump the following day, captured with their own helmet camera, before they filled out the same questionnaire. The analysis returned high correlations for emotions such as pleasure, engagement, and anger—but no correlation for fear. Interestingly, the participants reported low levels of fear immediately after the jump and also the day after—but their recorded heart rate told a different story. With almost no exceptions, the jumpers were close to their theoretical maximum heart rate before the jump. They had then been sitting still or performing only modest activity at the exit point for at least half an hour. One of the explanations for this lack of correlation may be found in the way we assess their emotional experiences.

#### Overall vs. Moment-to-Moment Self-Reports

Ordinary self-reporting measures are not designed to capture the intensity and variation experienced in activities such as BASE jumping. Our results show that standing on the top of the mountain before a jump is a highly unpleasant experience. During the jump, pleasure varies extensively, and after landing, the jumpers often report extreme levels of pleasure. Fear has a similar but inverse pattern. Therefore, asking the jumpers to rate the jump on a single-item Likert-like scale will only return a meaningless average for the duration of the experience. We found a similar pattern in a recent study of elite ski-jumpers [52]. To capture the experience of BASE jumping, we therefore tested out a new measure [5]. 

**Figure 1:**
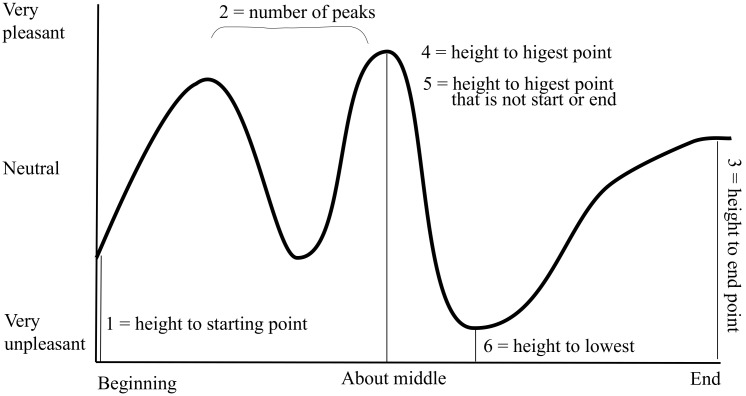
The Feelometer shows the six measuring points extracted in the study on BASE jumpers [5]. The *x*-axis represents the jump from before exit until after landing. The *y*-axis represents the magnitude of pleasure or interest, ranging from very unpleasant/uninteresting to very pleasant/interesting. Compressed example of the Feelometer used in the questionnaire, with the size of the *x*-axis being 11.5 cm and the size of the *y*-axis being 6.5 cm.

This instrument enables the participants to provide a retrospective moment-to-moment report from the episode. As shown in Figure 1, the *y*-axis shows the intensity of the emotions, and the *x*-axis is the timeline of the episode. The result is a schematic emotion report that gives the researcher the opportunity to make comparisons at different stages during the jump. We named it the Feelometer and extracted the following five measures: (1) height of starting point, (2) number of peaks, (3) height of end point, (4) height of highest point, and (5) height of lowest point. 

Such a graphical representation of the emotional experience allows the participants almost unrestricted possibilities when reporting their emotions. Although this captures more of the variety in an emotional experience, it poses great challenges when it comes to operationalizing and analyzing such data. In a later study on backcountry skiers, we therefore transformed this measure into seven questions, asking them to report their level of happiness, interest, and fear at seven different stages of the descent, from the top until after they stopped [6]. For a review on the challenges and risk in backcountry skiing, see [64,65].

## 3. How to Capture Momentary Emotions

Despite this complexity, one of the most common ways to measure emotions is by use of ordinary self-reports, or random repeated measures such as the experience sampling method (ESM) wherein people are cued—at random times—to report a current experience. Although most researchers recognize that such reports are prone to error, the ease of administering such measures still makes them the most frequently used measure of emotions and mental states. In addition to the inherent errors of self reports, several studies have demonstrated that cognitively reflecting over an experience might change the way people feel about them altogether [46]. The effect is similar to what is called re-appraisal, which is a well-known tactic to improve people’s experiences of negative events [66] but creates a challenge for researchers that aim to sample the emotional experience as it was experienced in the moment. Given these challenges, is there a way to tap into the flow of momentary and sometimes unconscious feelings without them being affected by the effort of measuring them?

Kahneman and Riis [67] have argued that, given that they are properly validated, physiological measures such as the EEG, heart rate, or skin conductance level would give an uninterrupted report of momentary emotions. However, at least in the case of action sport, which inherently involves physical activity, the activity itself will create a considerable amount of noise, rendering it impossible to disentangle the effect of psychological activity expressed physiologically from the mere physical activity itself. There are also issues of translating these measures into what they really mean in terms of experienced emotions, particularly in situations with ambiguity in bodily responses (e.g., misattribution of arousal, e.g., [68].

Functional MR studies are delivering exciting results, and they are able to pinpoint activity in brain areas associated with different emotional reactions. However, these measures are both costly, time-consuming and restricted to experiences that can be produced inside a three-foot-wide steel tube.

### 3.1. Facially-Expressed Emotions

Another way to measure momentary emotions is by observation and analyses of facial expressions. As the behavioral expressive component of emotions, facial expressions are (A) universal (however, there is evidence that suggests that people from East Asia express and interpret facial expression slightly differently compared to Western Caucasians, see, for example [68], and reliable indicators of discrete emotions which (B) co-vary with subjective experiences and (C) are a part of a coherent package of emotional responses that includes appraisals, physiological reactions, and other nonverbal behaviors and subsequent actions [69]. There is solid support for at least six universal facial expressions communicating happiness, fear, sadness, disgust, anger, and surprise [41]. However, new studies suggest that as many as seven additional positive emotions are associated with distinctive expressive displays. These emotions are: happiness, amusement, awe, contentment, interest, joy, love, and pride [70,71].

To perform a reliable measure of facial expressions, Ekman and Friesen [72] developed the facial action coding system (FACS), which is a way of directly measuring movements of the facial muscles. FACS consists of 46 action units, each representing an independent motion of the face, which in turn is combined into different facial expressions. A smile, for example, is characterized by an upward turn of the corners of the lips, which is produced by contraction of the zygomaticus major muscle [72] and described in the FACS system as the activation of the cheek raiser with action unit six (AU6) and lip corner puller (AU12). Sadness, on the other hand, is characterized by raised inner brows (AU1), lowered brow (AU4) and depressed lip corners (AU15). Facially expressed interest is characterized by activation of AU1 (inner brow raiser) and AU4 (brow lowerer) and also partially AU24 (lip pressor). Interestingly, the facial expression of interest as described by [71] bears no resemblance with happiness but a strong similarity with facially expressed sadness. In the past, the analysis of facial expression had to be performed manually. However, with advances in technology, this analysis can now be carried out with the help of software.

#### Automatic Coding of Facial Expressions

Automatic facial coding (AFC) has several advantages and a few disadvantages compared to manual coding. First and foremost, automatic coding demands very little labor. Recent advances in automatic coding of facial expressions have made this method more reliable and ubiquitous [73]. The software Facereader prodused by Noldus, Wageningen, Netherlandes, used for AFC either applies a direct FACS coding of facial movement or categorizes them into emotions. One major question is, of course, the validity and reliability of such tools. In other words: how well does the software actually measure what it is intended to measure? Lewinski and his colleagues [74] describe the validity and reliability of AFC based on (1) principle of computer algorithms, (2) psychological theories and (3) recognition studies. In software designed for scientific investigation, AFC build on psychological theories of human facial expression of emotions. These computer algorithms code facial expression after a given set of rules, which is applied to every image of a facial expression. Given large datasets of facial expression across ages, gender and ethnicities, such algorithms do not have any personal biases about gender, culture, or age. These algorithms are not prone to confirmation biases and are thereby truly blind to the condition.

FaceReader, which is the AFC application applied in the studies described in this paper, builds on Ekman’s [41] theory of universal facial expressions. FaceReader originally coded facial expression into categories of human emotion, but from version 6.0, FaceReader added functionality to code 24 different action units. In a reliability study, [74] tested FaceReader (version 6.0) against two publicly available and previously annotated datasets of human facial expressions: the Warsaw Set of Emotional Facial Expression (WSEFEP; 51) and the Amsterdam Dynamic Facial Expression Set (ADFES; 52). 

For facial expressions, FaceReader recognized 88% of the target emotions. The human emotion recognition for the same datasets was 85%. In the FACS analysis, FaceReader labeled the correct action units at 69% of the analyzed images. To receive a FACS certificate, a human coder must reach an agreement on 70%. Previous reliability studies testing the recognition of facial expressions of FaceReader version 1.0 have reported an accuracy of 89%. Similar results have been reported in other studies [75,76].

On the downside, the methods for coding facial expression are still being developed and refined, particularly by distinguishing different displays of positive emotions (see, for example, [71]). It takes time and effort before enough pre-coded material is available to update a computer model. In addition, humans are able to code head movements and body postures, which the software currently is unable to read. Therefore, happiness is still the only positive emotion included in the automatic analyses of facial expressions. In a study on mountain bikers, we tried to tease out facially expressed interest, but the results were inconclusive [8]. A major issue, though, is that the algorithms are trained on static images as also often applies for emotion recognition research in human participants, e.g., [32,75]. However, our emotions unfold over time [76].

Still, the advantages in most cases outweigh the cost, making this method increasingly popular, as seen in studies within the science of emotion [77,78], educational research [79,80], human–computer interaction [81], consumer behavior, user experience [82], clinical investigations of facial nerve grading in medicine [83], monitoring pain [81] and advertising and commercial films [84,85].

The rapid development of camera technology that has taken place in the past ten years is nothing less than a small revolution. These days, most of us is carrying a camera in our pocket with a quality that could produce a Hollywood movie [86]. Web cameras have improved in quality, and the development of small action cameras has made it possible to film from angles that were previously impossible—for example, capturing facially expressed emotions during an activity.

After testing the automatic facial coding technology in a study on tourists and their reaction to tourist commercial films, I realized that this might be the tool with which to capture emotions during a high-arousal activity [6]. Together with my colleagues, I developed a rig where we could mount a face-fronting action camera that could be carried during backcountry skiing.

**Figure 2:**
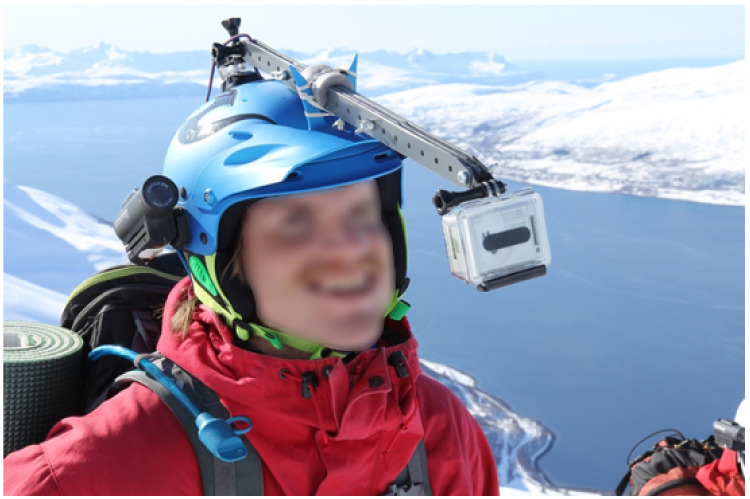
GoPro camera mounted on a ski helmet. This camera will capture the participant’s facial expressions during the activity. Most participants reported that they quickly habituated and paid little attention to the camera while skiing. Written informed consent has been obtained from the participant for the publication of this image.

However, mounting anything in front of the face in high-energy activities is dangerous, and the potential for serious harm to the participants should not be taken lightly. After months of testing, we eventually came up with the rig shown in Figure 2. Mounted this way, the camera will swing either up or sideways—away from the participant’s face—in case of a fall. If the camera is forced down towards the face, it will detach and fall off. We also added another camera capturing the participant’s point of view to be able to see the activity from their perspective. This made coding of the activity much easier. We have conducted several experiments with this rig, including a sample of backcountry skiers, mountain bikers, and ski jumpers [6,8,53].

## 4. How Are Momentary Feelings Transformed into Pleasant Memories?

In his seminal book from 1879, An Introduction to the Principles of Morals and Legislation, Bentham [87] described a method for calculating the value of pleasure and pain. This has come to be known as the hedonimeter. Bentham further articulated the doctrine of ethical hedonism, which claims that pleasure is the only good and pain the only bad. Hence, human goodness is simply the accumulation of pleasant moments. Bentham further argued that the value of pleasure could potentially be measured according to its intensity and duration. This idea was further developed by Kahneman, who argued that this might be solved by the use of ecologically valid self-reports in combination with sophisticated methods such as the assessment of physiological indicators of hedonic states [88]. According to the logic of hedonism, this procedure offers data that are unaffected by memory and evaluation biases and should grant insights into the participants’ momentary emotions, or what Kahnemann called “true” feelings, or objective happiness [88].

Although Kahneman supported the idea that feelings can be measured moment-by-moment, he never argued that adding these momentary feelings were equivalent to the mental image that we store in memory representing this experience. Rather, he introduced a distinction between momentary feelings (the experiencing self) and memories of that experience (the remembering self). 

Two factors seem important when emotional episodes evolve from experiences to memories. The first is emotional states at key points such as the beginnings, endings, and during emotional peaks or troughs [67,76,89]. The second reflects the slope or emotional development over time—for example, if happiness is increasing or decreasing [90,91]—and the rate of change—how fast things are improving or deteriorating [92,93]. Features such as the duration of the experience have been found to have little or no impact on the retrospective evaluations [94]. Following this logic, the memory of an experience should be predicted on the basis of how it started and ended, as well as its peaks, troughs, and/or emotional trajectory during the experience.

Although numerous studies verify the impact of these key moments, some factors might moderate or eliminate the effect. First, people might have many similar experiences to the one studied, and thus have experienced more intense peaks in a time before the study or experience several peaks with nearly similar intensity within the study and, as such, render the measured peaks as less important [84,91]. Second, the extreme effect of peaks fades more rapidly over time, causing the peaks to be recalled less intensely [95] and thus flattening the emotional profile.

In addition, the study of how such defining features affect the overall evaluation of entire events has focused mainly on single episodes with a defined beginning and end. Ariely and Zauberman [96] have demonstrated that if a single episode is broken down into different subparts, the impact of improving trends, or slopes on overall evaluations, is significantly reduced.

### Momentary: Facially-Recorded Emotions vs. Overall Self-Reported Emotions

In our studies on backcountry skiers and downhill bikers, we found a large discrepancy between momentary and retrospective emotional experiences [6,8]. In a recent mixed-model study on elite ski-jumpers [53], we found that the participants described a somewhat similar overall emotional profile in qualitative interviews and retrospective self reports. However, there is also a discrepancy between the qualitative interviews and quantitative measures and the self-reported emotions and moment-to-moment facially expressed emotions.

One difference between the self-reported and facially expressed emotions is the lack of happiness during the ski jump. The participant describes and reports feeling joy during flight, but facially expressed happiness only surfaces after landing. Interestingly, when we asked the participants to report their expected emotional experiences, they all—presumably based on experiences from the past—described that they expected the jump to be more joyful and less fearful than they reported the actual jump to be immediately after. It thus seems that the closer we get to the actual jump, the less happiness or joy we find. 

In the early phases of the jump, the participants describe and report feeling a mix of fear, excitement, and being completely immersed. There is currently no facial expression for excitement or interest built into the FACS system, even though Campos and colleagues [71] have described it along with several other facially expressed positive emotions. Instead, the corresponding moment-to-moment measures returned large sections that the AFC labeled disgust, and also sadness, in addition to the expected fear. These readings might be explained by taking a closer look at the instrument itself and some inherent challenges in capturing facially expressed emotions in the field. 

## 5. Limitations and Future Research

The method of filming and analyzing facially expressed emotions in the field described in this study opens up new possibilities for uninterrupted measures of emotions during experiences. However, the method is not without shortcomings. The technology was developed to work in a controlled laboratory setting and a shift in light conditions may impact the quality of the reading. To obtain a good reading of facially expressed emotions, the participants can not wear any eye protection. This might be a challenge in some activities such as backcountry skiing. The camera also needs to be mounted in the participant’s field of vision. Even though the participants say they quickly habituated and did not pay much attention to the camera, great care needs to be taken in order to produce a setup in which the camera will not come lose and harm the participant. This should not be taken lightly.

Measuring emotions is a challenging task regardless of the method. According to Hannais and Ekkekakis [54], as much as 80–85% of the self-generated emotion labels are not included in the various standardized emotion scales. For automatic analysis of facially expressed emotions, the list of emotions to read is even shorter. In addition, there might be challenges in the coding of facial expressed emotions itself.

### Potential Error in Coding of Facially Expressed Emotion

In the study of BASE jumpers, one of the interesting findings was that the participants reported no sadness at all [5]. However, when we recorded the facial expressed emotions to a sample of tourists while they watched tourist commercial films, they displayed more sadness than happiness [85]. Among backcountry skiers, we found sadness to be the second most prominent emotion after happiness. 

A possible explanation may lie in the action units that are the building blocks in FACS. Interest and sadness share two action units; raised inner brows (AU1) and lowered brows (AU4). Interest also activates compressed lips (AU 24), whereas sadness is characterized further with depressed lip corners (AU15) instead of compressed lips. 

In a physically strenuous activity such as backcountry skiing, the participants, more often than not, breathe through their mouth and not their nose. An open mouth is incompatible with compressed lips and maybe also with depressed lip corners. This leaves little difference between a sad and an interested facial expression. We therefore speculated that the relatively high levels of facially expressed sadness may partly be mislabeled interest. This would mirror the predictions from the Functional Wellbeing Approach. However, in a follow up study in which we applied the same method on a sample of mountain bikers, we did not find the same high levels of sadness [8].

So, why is interest not one of the basic emotions described by Ekman? The answer is coincidence more than anything else. In an interesting paper, Ellsworth [97], who, at the time of the development of the FACS system, worked alongside Ekman and Friisen, describes how they needed pre-coded pictures to develop the tools to test if facially expressed emotions were universal. They were working towards a deadline for the journey to New Guinea and the first of what should be followed by a large series of replication studies. They simply ran out of time and had to go with what they had. For some emotions, they were only an image short. Interest was definitely on the list, and Caroll Izard [98], who was carrying out cross-cultural studies at the time, found support for several additional emotions, including interest. So, an image short in the late 1960s, 60 years later, unfortunately, leaves us shorthanded when it comes to capturing more of the moment-to-moment dynamics in intense emotional experiences in extreme sport.

Future research should validate the findings with low levels of happiness during intense activities such as extreme sport and also applying different methods for capturing and comparing emotions during and after an activity.

## 6. Conclusions

So, how was it? It much depends on when and how you ask. Intense experiences are probably not as pleasant or joyful in the midst of it as you remember. However, the memory of such events can be a shining beacon guiding our path into the future. To fully understand the experience, we need to know not just to know how it was, but how it is. The current paper discusses some challenges with measuring emotions in extreme sport in the field and also presents a recently developed method where the participants facially expressed emotions can be captured with a camera and analyzed in post. This method provides a glimpse into how extreme-sport activities are experienced in the midst of it. When compared to emotional assessments in post, this may be able to explain why many extreme-sport participants report happiness to be among the key motivations for taking part, even though they apparently do not express much happiness during the activity itself.

## Data Availability

This article elaborates on previous published studies. Please refer to the original studies.
